# Elucidation of the compatible interaction between banana and *Meloidogyne incognita* via high-throughput proteome profiling

**DOI:** 10.1371/journal.pone.0178438

**Published:** 2017-06-02

**Authors:** Aisyafaznim Al-Idrus, Sebastien Christian Carpentier, Mohamad Taufiq Ahmad, Bart Panis, Zulqarnain Mohamed

**Affiliations:** 1 Genetics and Molecular Biology Division, Institute of Biological Sciences, Faculty of Science, University of Malaya, Kuala Lumpur, Malaysia; 2 Center for Research in Biotechnology for Agriculture (CEBAR), University of Malaya, Kuala Lumpur, Malaysia; 3 Laboratory of Tropical Crop Improvement, Division of Crop Biotechnics, Faculty of Bioscience Engineering, Katholieke Universiteit Leuven, Leuven, Belgium; 4 SYBIOMA: Facility for SYstems BIOlogy based MAss spectrometry, Leuven, Belgium; 5 Bioversity International, Belgian Office at KU Leuven, Leuven, Belgium; Bhabha Atomic Research Centre, INDIA

## Abstract

With a diverse host range, *Meloidogyne incognita* (root-knot nematode) is listed as one of the most economically important obligate parasites of agriculture. This nematode species establishes permanent feeding sites in plant root systems soon after infestation. A compatible host-nematode interaction triggers a cascade of morphological and physiological process disruptions of the host, leading to pathogenesis. Such disruption is reflected by altered gene expression in affected cells, detectable using molecular approaches. We employed a high-throughput proteomics approach to elucidate the events involved in a compatible banana- *M*. *incognita* interaction. This study serves as the first crucial step in developing natural banana resistance for the purpose of biological-based nematode management programme. We successfully profiled 114 Grand naine root proteins involved in the interaction with *M*. *incognita* at the 30^th^- and 60^th^- day after inoculation (dai). The abundance of proteins involved in fundamental biological processes, cellular component organisation and stress responses were significantly altered in inoculated root samples. In addition, the abundance of proteins in pathways associated with defence and giant cell maintenance in plants such as phenylpropanoid biosynthesis, glycolysis and citrate cycle were also implicated by the infestation.

## Introduction

Plants are constantly exposed to a range of pathogenic organisms inhabiting the soil. Amongst these, plant-parasitic nematodes (PPN) are documented as soil pathogens of economic importance incurring approximately US$100 billion worth annual crop losses [1]. Amongst the PPN, sedentary root-knot nematodes (RKN; *Meloidogyne* spp.) are one of nature’s most successful obligate parasites. *Meloidogyne incognita* was reported to be the most widely distributed species in this genus attacking more than 3000 plant species including agricultural crops [2, 3, 4, 5, 6] such as bananas (*Musa* spp.). Banana production was considerably affected by RKN infestations particularly in the absence of *Radopholus similis* [7, 8]. RKN are a problem for bananas in the tropics especially in Asian countries [8] and in dry sub-tropical countries [9]. At least five RKN species infect *Musa* with *M*. *incognita* and *M*. *javanica* being the more commonly found [10, 11, 12].

Similar to other RKN species, *Meloidogyne incognita* is a sedentary plant-parasite with evolved strategies to infest plant species by manipulating fundamental key elements of plant cell development [13]. During a compatible interaction, this parasite induces re-differentiation of root cells into multinucleated nematode feeding cells (giant cells). These giant cells operate as nutrient sinks that meet the nutritional demands of developing nematode individuals. Synchronously, hyperplasia and hypertrophy of the surrounding cells lead to the formation of a root gall [13, 14], thus affecting plant growth and its anchorage system. Detailed infection mechanism and feeding site establishment process have been reported/reviewed in [15, 16, 17, 18, 19]. Wilting and stunted growth are distinctive symptoms for infected banana plants [20], leading further reduction in bunch weight and fruit production. Jonathan et al. [21] reported that banana fruit size, total carbohydrate, total soluble sugars and ascorbic acid were reduced as a result of *M*. *incognita* infestation. As such, there is a dire need for effective control of this nematode in banana farms.

Control measures at the macro level such as crop rotation and using clean planting materials are inefficient while the only effective solution using nematicides is undesirable due to its detrimental effect to the humankind and the environment [22]. Therefore, alternative management strategies such as crop genetic improvement are gaining new interest worldwide [8]. Several nematode resistance (*Nem-R*) genes expressing NBS-LRR proteins [23] have been isolated from various plants [24, 25], all conferring resistance against sedentary endoparasites [24]. The first nematode resistance gene cloned was *Hs1*^*pro-1*^ from sugar beet conferring resistance against sugar beet cyst nematodes [26, 27]. Four other genes namely *Mi-1*, *Hero A*, *Gpa2* and *Gro1-4* were cloned from tomato and potato relatives [24]. However since a gene transfer attempt from tomato into tobacco using the cloned *Mi* gene was unsuccessful [27], efforts were focused more on searching for plants’ own natural tolerance/resistance gene(s) against the pathogen. Therefore the quest for new mechanisms acting against *M*. *incognita* relies primarily on the knowledge garnered from plant-pathogen interaction studies. Since there is, as yet, no resistant banana cultivar reported against *M*. *incognita*, the current work describes our attempt to model the compatible interaction with *M*. *incognita* using the commercial dessert cultivar, Grand naine.

Parasite survival strategies and host defence mechanisms are the key factors governing the complex host-nematode relationship [18]. For the past 18 years, many studies utilising different approaches have been conducted to obtain deeper understanding of plant-pathogen interaction in order to formulate efficient control strategies [1, 18, 28, 29]. In the advent of the ‘omics’ era, proteomics is seen as a potential platform to ‘decode’ the communication used between the two organisms due to the fact that proteins are the most immediate macromolecules involved in biological actions and responses. A comprehensive review on proteomic analysis of plant response to nematode infection can be obtained in Escobar et al. [30]. Echoing that of mentioned in Palomares-Rius et al. [31] and Quirino et al. [32], we found that most proteomic analyses of plants subjected to biotic and abiotic treatments were conducted on plant model species such as Arabidopsis and rice. Besides, amongst a handful of proteomics studies on plant-pathogen interactions, most of them were carried out to address bacterial and fungal diseases of foliar tissues and not many of root tissues.

The difficulty in carrying out proteomics studies on root-pathogen interactions has been reported in a review [33]. Recently, Palomares-Rius et al. [31] and Li et al. [34] reported a commendable 2D-based proteomic study of tripartite interaction on chickpea and Fusarium-banana root interaction, respectively. However, the throughput of gel-based systems is limited and only a scarce amount of interesting proteins has been discovered. Therefore we adopted the gel-free, Liquid Chromatography-Mass Spectrometry (LC-MS)—based Proteomics platform to elucidate proteins involved in banana-*M*. *incognita* interaction at the 30^th^- and 60^th^—day after inoculation (dai). The two time points were chosen in order to obtain an increased number of galled cells for increased peptide abundance detection. Gel- and label-free LC-MS methods represent attractive alternatives since they are robust and amenable to all types of biological samples, despite being cost effective. This is the first high throughput report to reveal the molecular profile and dynamic changes occurring in banana roots inoculated with *M*. *incognita*. Our report adds new insights to plant reactions during nematode infestation and new knowledge on the weak points in plant defence mechanisms which can serve as the basis towards developing a molecular-based nematode combating regime.

## Materials and methods

### Plant materials and *M*. *incognita* culture

Banana cv. Grand naine (ITC 1256) plantlets were obtained from the International Transit Centre (ITC) of *Musa* collection at Katholieke Universitiet Leuven, Belgium. The plantlets were propagated and maintained in Murashige and Skoog (MS) basal media (pH 5.8) supplemented with 4.1 μM biotin, 5.7 μM indoleacetic acid (IAA), 5.4 μM naphtaleneacetic acid (NAA), 87 μM sucrose and 2 g/L gelrite with 3 mg/L 6-Bensylaminopurine (BAP) prior to transfer onto rooting media (MS including vitamins, 1 mL/L ascorbic acid, 30 g/L sucrose, 2 g/L gelrite and 0.5 g/L active charcoal, pH 6.15 at 60°C). Banana plantlets were left in the rooting media for a month until reaching a four or five-leaf-stage prior to transplantation. Transplantation and acclimatisation processes were carried out as described in [35]. Briefly, the plantlets were transplanted into a 200 mL plastic pots containing sand: peat (2:1) soil mixture and left to acclimatise under greenhouse conditions for 8 weeks. Fertiliser was applied twice a week starting from the second week post transplantation. The plants were later transferred into 1 L pots for inoculation procedure. *Meloidogyne incognita* (Malaysian population) culture was obtained from Malaysian Agricultural Research and Development Institute (MARDI). This culture was then propagated and maintained in Grand naine host plants until future use.

### Nematode inoculation

Collection of RKN egg masses from plant hosts and hatching of juvenile stage two (J2) nematodes were carried out as described in [35]. To estimate the number of J2 used for treatments, J2 individuals obtained per mL were counted in triplicates and later averaged. dH_2_O was used to adjust to 1000 J2 per treatment. Treatment was carried out using a single inoculation protocol as we previously described [35]. Briefly, plants transplanted into 1 L pots were left to acclimatise for a week. Following that, one of the primary roots was selected as an inoculation target. The selected root was placed across a 3.5 cm diameter plastic Petri dish (the target root fragment) and left in soil for three days prior to inoculation. One thousand J2 nematodes were inoculated onto the targeted root fragment and the target site was left in soil until the experiment was terminated at either 30- or 60-days after inoculation (dai). Each time point consisted of three nematode-inoculated banana plantlets and three control plantlets (three biological replicates for each treatment). Upon harvesting, the targeted inoculation site was excised from the root system (one root fragment for each banana plant), thoroughly washed under running tap water, air-dried and weighed. The weighed root fragment was placed into a fresh 1.5 mL microcentrifuge tube, snap-frozen in liquid nitrogen and then stored in -80°C until further lyophilisation.

### Root tissue lyophilisation and protein isolation

Stored banana root sample was lyophilised for 48 hours at -100 C° according to [36]. Banana root proteins were then extracted using phenol extraction methanol/ammonium acetate precipitation-based protocol as described in [37] with minor modifications suitable for LC-MS shotgun platform. Protein extraction procedure was conducted at 4°C unless mentioned otherwise. Briefly, 60 mg lyophilised banana root tissue were ground using cooled mortar and pestle in the presence of liquid nitrogen and later suspended in a fresh 2 mL microcentrifuge tube containing 750 μL Extraction buffer [100 mM Tris-HCl pH 8.3, 5 mM EDTA, 100 mM KCl, 1% w/v DTT, 30% w/v sucrose; complete protease inhibitor cocktail (Roche Applied Science) and mixed. Subsequently, 750 μL buffered phenol were added to the sample and vortexed for 10 min. The mixture was then centrifuged (12,000 rpm, 10 min) and the resulting phenolic phase was transferred into a fresh microcentrifuge tube and protein was re-extracted using equal volume of extraction buffer. Sample was then centrifuged (12, 000 rpm, 5 min) and resulting phenolic phase was transferred into a fresh tube. Proteins were precipitated overnight in 5 volumes methanol (100mM ammonium acetate) at -20°C. Following this, the sample was then centrifuged (13,000 rpm, 60 min) to collect the pellet and later rinsed twice in 2 mL rinsing solution (cold acetone/0.2% DTT). Note that for the first rinse, the pellet was left in the rinsing solution for 1 hr at -20°C and centrifuged (13, 000 rpm, 30 min). Cleaned pellet was left air-dried and suspended in 100 μL Lysis buffer (8M urea, 5mM DTT, 30 mM Tris). Protein quantification was carried out using 2-D Quant Kit (Amersham, UK) following manufacturer’s protocol.

### Peptide digestion and separation

Twenty micrograms of protein sample was incubated in 0.02 M DTT for 15 min. This mixture was then mixed with 0.05 M iodoacetamide and incubated in the dark for 30 min prior to dilution in 0.05 M ammonium bicarbonate. An aliquot of 20 μg of protein was then digested with 0.2 μg/μL trypsin and incubated overnight at 37°C. Samples were acidified with trifluoroacetic acid (0.1% final concentration) and de-salted using C18 solid phase extraction according to the manufacturer (Pierce^™^ C18 Spin Columns, Thermo Fisher Scientific, Ghent, Belgium). Peptides were eluted with 40 μL 70% acetonitrile (ACN), after which solvents were evaporated using a speedvac and dissolved in 5% ACN, 0.1% formic acid.

### Peptide separation and MS analysis

The UPLC—MS/MS analysis was performed on a Q Exactive Orbitrap mass spectrometer (Thermo Scientific, USA) as described by [38]. The samples (5 μL containing 1 μg peptides) were injected and separated on an Ultimate 3000 UPLC system (Dionex, Thermo Scientific) equipped with a C18 PepMap100 pre-column (5 μm, 300 μm × 5 mm, Thermo Scientific) and an EasySpray C18 column (3 μm, 75 μm × 15 cm, Thermo Scientific) using a gradient of 5% to 20% ACN in 0.1% formic acid (FA) in 10 min followed by a gradient of 10% to 35% ACN in 0.1% FA in 4 min and then a final gradient from 35% to 95% ACN in 0.1% FA in 2.5 min. The flow-rate was set at 250 μL/ min. The mass spectrometer was operated in a positive ion mode with a nanospray voltage of 1.5 kV and a source temperature of 250°C. ProteoMass LTQ/FT-Hybrid ESI Pos. Mode CalMix (MSCAL5-1EA SUPELCO, Sigma-Aldrich) was used as an external calibrant and the lock mass 445.12003 as an internal calibrant. The instrument was operated in a data-dependent acquisition (DDA) mode with a survey MS scan at a resolution of 70,000 (FWHM at m/z 200) for the mass range of m/z 350–1800 for precursor ions, followed by MS/MS scans of the top 10 most intense peaks with + 2, + 3 and + 4 charged ions above a threshold ion count of 16,000 at a 35,000 resolution using a normalised collision energy (NCE) of 29 eV with an isolation window of 3.0 m/z and dynamic exclusion of 10 s. All data were acquired with Xcalibur 2.2 software (Thermo Scientific).

### Protein identification

All raw data were converted into mgf files using Progenesis v4.1 (Nonlinear Dynamics, UK). The spectra were searched using Mascot (Version 2.2.06; Matrix science, London, England) against our in-house *Musa* database (76,220 sequences) containing all protein sequences of published A and B genome along with contaminant sequences (trypsin and keratin). The following parameters were used: the enzyme was trypsin and one miscleavage was allowed, cystein-carbamidomethylation was chosen as a fixed modification and methionine-oxidation as a variable one. Precursor peptide charge state was 2+ and 3+, error window on experimental peptide mass values was 10 ppm and 20 absolute milli-mass units were chosen for fragment ion mass tolerance. Peptides assigned to keratin or trypsin were not taken into account.

For data validation, the false discovery rate (FDR) was calculated using Scaffold (Version: Scaffold_3.6.3; Proteome Software Inc., Portland, OR, USA). An integrated version of X! Tandem (Version: CYCLONE, 2010.12.01.1) in Scaffold was used for an additional database searching. For FDR calculation, the search results from both Mascot and X! Tandem were combined automatically by Scaffold with the following settings: a peptide confidence level of 95%, a protein confidence level of 80%, a minimum peptide number of 1, and the thresholds of each search engines separately.

### Peptide quantification and data analysis

Quantitative analysis was performed using Progenesis LC—MS version 4.1 (Nonlinear Dynamics) as described by [39] with some modifications. For alignment, a reference run was selected, after which the files were aligned automatically, manual landmark vectors were not necessary. The sensitivity of the peak picking limits was put to four. With these settings, Progenesis LC—MS generates an aggregate run that contains all ions from the analysed runs. Peptides with charges two to five were retained in the filter step, and then the data were normalised by calculating abundance ratios to a reference run. Peptides with p-value < 0.05 (Progenesis) were retained for further analysis and abundances of all corresponding significant peptides were summed per protein accession number. By this, protein abundances were calculated based on peptide abundances. A list of identified proteins was then generated and subjected to Principal Component Analysis (PCA) to assess whether or not proteins from similar experimental condition cluster with each other. The software STATISTICA (Version 8.0; Tulsa, OK, USA) was used for data analysis. Statistical evaluation started with a principal component analysis (PCA) according to [40]. Only proteins (ANOVA: P≤0.05) with more than 1.5 times fold change and showing maximum ion score of more than 30 were deemed significant and selected for further analysis. For ease of analysis, pairwise comparison matrices were used to compare between treatments i.e 60-dai inoculated vs. 60-dai control samples; and 30-dai inoculated vs. 30-dai control samples in order to profile proteins involved during the interaction. For these matrices, protein abundance in control samples were used as the baseline to obtain protein fold changes. These proteins were then subjected to Gene Ontology (GO) analysis using BLAST2GO and grouped based on GO levels obtained from this software. In cases where a protein was present with more than one GO level, the most general level that described its function was chosen. Protein-GO interactions were mapped using Cytoscape (available for free at http://www.cytoscape.org/download.php).

## Results

### Banana root proteins showed significant abundance difference upon interaction with *Meloidogyne incognita*

**Fig 1:**
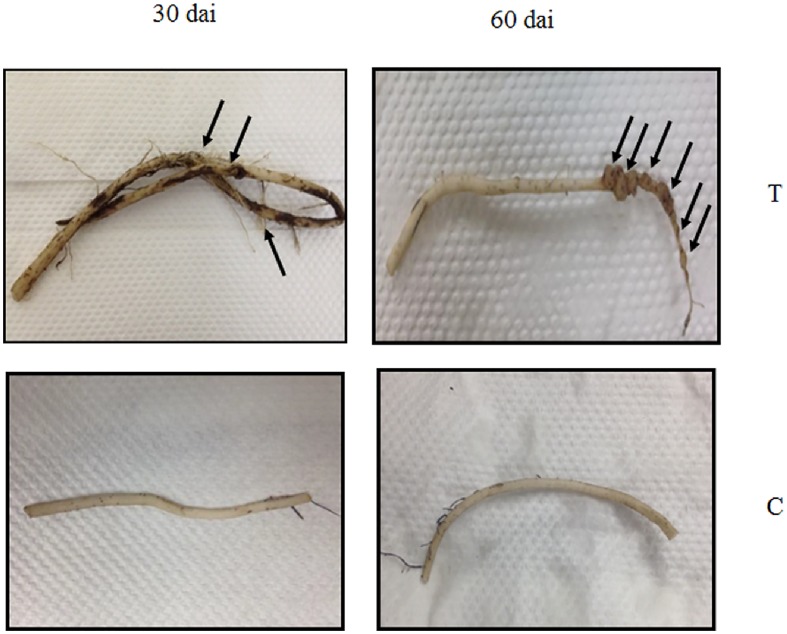
An example of severity of galls observed on the 60^th^-dai compared to the 30^th^-dai that were visible to the naked eye, indicated by the arrows. No galls formed in control root fragments for both time points.

**Fig 2:**
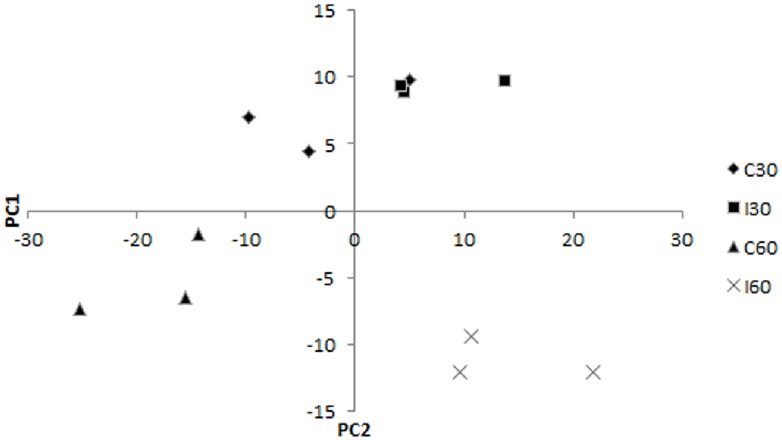
Principle Component Analysis (PCA) conducted on 408 proteins showed PC1 separates 60-dai inoculated samples (I60) from 60-dai control samples (C60) while definitive separation was not obtained for 30-dai inoculated samples (I30) and 30-dai control samples (C30). On the other hand, PC2 separates 30-dai samples (C30 and I30) from 60-dai samples (C60 and I60).

In this study, we observed that galls were more visible to the naked eye as swellings on inoculated banana roots at 60-dai compared to root fragments inoculated for 30 days (Fig 1). No galls were present in all control samples. A total of 408 proteins were subjected to Principal Component Analysis (PCA). We found that principal component one (PC1) separated 60-dai control samples from 60-dai inoculated samples. However, definitive clustering was not obtained for samples harvested at 30-dai (Fig 2). On the other hand, all 30-dai samples were separated from the 60-dai samples by PC2. We identified 114 root proteins showing significant differential abundance levels between nematode-inoculated root tissues and control root tissues at 30-dai and 60-dai (Table 1).

**Table 1:** List of proteins showing significant abundance difference (together with their accession numbers) in inoculated samples at 30- and 60- dai after inoculation with 1000J2 *M*. *incognita*. The list is sorted according to the fold change of each protein.

Protein	Reference No.	Fold change	AnovaP ≤0.05	Max ion score	No. of peptides
Fundamental Biological Processes					
60-dai					
40s ribosomal protein s2-3-like	GSMUA_AchrUn_randomP09450_001_MUSAC	Detected only in inoculated	0.01	34.37	1
60s ribosomal protein I22-2-like	GSMUA_Achr3P00720_001_MUSAC	Detected only in inoculated	0.01	54.86	1
60s ribosomal protein l10a-1-like	ITC1587_Bchr3_P07546_MUSBA	Detected only in inoculated	0.01	58.35	3
Adenosine kinase 2-like	GSMUA_Achr2P00250_001_MUSAC	Detected only in inoculated	0	51.3	3
Cinnamyl alcohol dehydrogenase	GSMUA_Achr4P06150_001_MUSAC	Detected only in inoculated	0	32.51	5
60s ribosomal protein l9-like	ITC1587_Bchr5_P13916_MUSBA	469.7	0.01	47.46	2
40s ribosomal protein s20-2-like	ITC1587_Bchr3_P06192_MUSBA	55	0.02	33.43	2
40s ribosomal protein s15a-1	GSMUA_Achr1P17170_001_MUSAC	29.4	0.01	38.51	1
40s ribosomal protein s16-like	ITC1587_Bchr6_P15714_MUSBA	27.3	0	34.19	1
40s ribosomal protein s14	GSMUA_Achr2P20380_001_MUSAC	22	0.01	53.54	2
S-adenosylmethionine synthase	ITC1587_Bchr7_P18740_MUSBA	17	0.03	30.77	2
V-type proton ATPase catalytic subunit a-like	GSMUA_Achr11P08060_001_MUSAC	3.5	0.05	79.24	1
40s ribosomal protein s4-3	GSMUA_Achr2P01640_001_MUSAC	3.4	0.03	46.34	2
S-adenosylmethionine synthase 2	ITC1587_Bchr1_P01149_MUSBA	3.4	0.01	34.37	1
60s ribosomal protein l4-like	GSMUA_Achr5P03060_001_MUSAC	3.2	0.01	43.69	5
40s ribosomal protein sa-like	GSMUA_Achr1P21820_001_MUSAC	2.4	0.02	33.36	3
ATP synthase f0 subunit 1	GSMUA_AchrUn_randomP15230_001_MUSAC	-1.5	0.03	55.78	6
ATP synthase subunit	GSMUA_Achr10P27350_001_MUSAC	-1.6	0.01	74.99	1
ATP synthase subunit	GSMUA_Achr9P21710_001_MUSAC	-2	0.01	82.42	8
Probable ATP synthase 24 kda (mitochondrial)	GSMUA_Achr6P02850_001_MUSAC	-2.2	0	54.84	2
ATP synthase subunit mitochondrial	ITC1587_Bchr10_P31293_MUSBA	-2.6	0.01	31.84	1
Aspartate (mitochondrial)	ITC1587_Bchr6_P16093_MUSBA	-2.4	0	87.65	2
Phosphoenolpyruvate carboxylase	GSMUA_Achr6P26850_001_MUSAC	-2.6	0	33.06	1
Aspartate-semialdehyde dehydrogenase	GSMUA_Achr10P18110_001_MUSAC	-3.6	0.022	89.03	1
Pyruvate dehydrogenase e1 component subunit beta- (mitochondrial)	GSMUA_Achr5P25000_001_MUSAC	-3.7	0.02	74.19	4
Minor allergen alt a 7-like	GSMUA_Achr5P26440_001_MUSAC	-6.1	0.02	34.57	2
Binding					
60-dai					
General					
Probable calcium-binding protein cml7	ITC1587_Bchr9_P27746_MUSBA	5.2	0.05	40.13	1
Germin-like protein 5–1	GSMUA_Achr5P18440_001_MUSAC	-7.5	0.01	32.5	1
RNA					
Elongation factor 1-alpha	ITC1587_Bchr6_P15150_MUSBA	Detected only in inoculated	0	51.4	4
Elongation factor 2	GSMUA_Achr4P01020_001_MUSAC	Detected only in inoculated	0	38.36	5
Protein					
O-methyltransferase	ITC1587_Bchr3_P07963_MUSBA	Detected only in inoculated	0.04	35.39	2
Carbohydrate					
Protein gos9-like	ITC1587_Bchr9_P25965_MUSBA	73.6	0.02	37.89	1
Small molecule					
Succinate dehydrogenase	GSMUA_Achr6P31640_001_MUSAC	-2.8	0.01	48.27	2
Succinate dehydrogenase	ITC1587_Bchr7_P18621_MUSBA	-2	0.01	60.71	5
Biosynthetic and primary metabolic process					
30-dai					
Biosynthetic and primary metabolic process					
Caffeoyl- O-methyltransferase	GSMUA_Achr6P36400_001_MUSAC	5.3	0.04	33.56	2
60-dai					
Biosynthetic process only					
Alpha-glucan-protein synthase	ITC1587_Bchr4_P10810_MUSBA	3.7	0.03	52.64	5
Biosynthetic and primary metabolic process					
Aspartate (cytoplasmic)	GSMUA_Achr4P08110_001_MUSAC	Detected only in inoculated	0.05	61.97	2
5-methyltetrahydropteroyltriglutamate—homocysteine methyltransferase 1	ITC1587_Bchr5_P11892_MUSBA	19.8	0.03	39.61	3
5-methyltetrahydropteroyltriglutamate-homocysteine expressed	ITC1587_Bchr4_P10741_MUSBA	16.3	0	72.27	2
5-methyltetrahydropteroyltriglutamate—homocysteine methyltransferase	GSMUA_Achr7P01530_001_MUSAC	5.4	0	48.51	8
5-methyltetrahydropteroyltriglutamate-homocysteine expressed	GSMUA_Achr4P22700_001_MUSAC	3.2	0	73.39	5
5-methyltetrahydropteroyltriglutamate—homocysteine methyltransferase	GSMUA_Achr4P21470_001_MUSAC	2.9	0.03	57.16	3
Methylthioribose kinase-like	GSMUA_Achr7P05460_001_MUSAC	-4.4	0.05	57.09	2
Cysteine synthase	ITC1587_Bchr4_P10620_MUSBA	-5.2	0.01	46.61	2
Catabolic process					
30-dai					
Peroxidase 4-like	ITC1587_Bchr11_P34142_MUSBA	-3.9	0.04	58.08	1
60-dai					
Methylmalonate-semialdehyde dehydrogenase	GSMUA_Achr4P22360_001_MUSAC	-3.3	0.04	59.01	1
Probable aldehyde dehydrogenase isoform x1	GSMUA_AchrUn_randomP11080_001_MUSAC	-5.8	0.01	43.62	3
Monodehydroascorbate (chloroplastic)	GSMUA_Achr5P17510_001_MUSAC	-15.3	0	30.18	3
Catalytic and hydrolytic activity					
60-dai					
Ras-related protein raba5c-like	ITC1587_Bchr11_P33367_MUSBA	Detected only in inoculated	0	48.91	4
UDP-glucuronic acid decarboxylase 6-like	GSMUA_Achr6P05080_001_MUSAC	4.4	0	46.94	3
Lignin-forming anionic peroxidase-like	GSMUA_Achr4P05250_001_MUSAC	-4.1	0.01	95.8	7
Biotin carboxylase (chloroplastic)	ITC1587_Bchr8_P24200_MUSBA	3	0.04	47.45	1
Heat shock protein 70	GSMUA_Achr2P16250_001_MUSAC	-4.9	0.02	57.06	4
Probable plastid-lipid-associated protein (chloroplastic)	GSMUA_Achr4P20110_001_MUSAC	-6	0	36.97	1
Cellular component organization					
60-dai					
Eukaryotic translation initiation factor 5a-2	GSMUA_Achr3P18790_001_MUSAC	Detected only in inoculated	0	38.92	2
Isocitrate dehydrogenase	GSMUA_Achr1P05110_001_MUSAC	Detected only in inoculated	0.01	43	4
Tubulin beta chain-like	ITC1587_BchrUn_random_P35428_MUSBA	Detected only in inoculated	0	51.4	1
Tubulin beta chain (isoform 2)	ITC1587_Bchr6_P16601_MUSBA	200.8	0	62.54	1
Tubulin alpha-3 chain-like	ITC1587_Bchr6_P17875_MUSBA	14.2	0.03	52.63	2
Actin-101-like	GSMUA_Achr10P03730_001_MUSAC	-1.8	0.05	83.52	1
Tubulin beta chain (isoform 1)	GSMUA_Achr6P04600_001_MUSAC	-201.5	0.01	40.93	1
Cellular and primary metabolic process					
60-dai					
Serine hydroxymethyltransferase 4	ITC1587_Bchr9_P25209_MUSBA	Detected only in inoculated	0.01	88.2	2
Glyceraldehyde-3-phosphate dehydrogenase (cytosolic-like)	GSMUA_Achr5P25410_001_MUSAC	468.9	0.01	61.23	2
Sucrose synthase 2-like	GSMUA_Achr6P10890_001_MUSAC	7.3	0.03	54.32	5
Pyruvate cytosolic isozyme-like	GSMUA_Achr10P15400_001_MUSAC	5.2	0.05	41.3	4
Pyruvate cytosolic isozyme	ITC1587_Bchr2_P03452_MUSBA	2.1	0.01	43.52	2
Peroxiredoxin (mitochondrial)	GSMUA_Achr8P09520_001_MUSAC	-2.6	0.03	37.61	1
Establishment of localisation					
60-dai					
Guanosine nucleotide diphosphate dissociation inhibitor 2	GSMUA_Achr6P18380_001_MUSAC	-3.0	0.03	33.95	1
Homeostatic process					
60-dai					
cbs domain protein	ITC1587_Bchr3_P07894_MUSBA	-2.2	0	71.38	1
Hydrolytic activity					
60-dai					
3-hydroxyisobutyryl- hydrolase-like protein (mitochondrial)	GSMUA_Achr6P00740_001_MUSAC	-2	0.04	60.62	2
Metabolic process					
60-dai					
Carbohydrate					
Fructose-bisphosphate aldolase cytoplasmic isozyme-like	ITC1587_Bchr5_P14394_MUSBA	-5.1	0.02	82.46	3
Fructose-bisphosphate aldolase	ITC1587_Bchr8_P21572_MUSBA	Detected only in inoculated	0.01	95.68	4
Macromolecule					
Probable fructokinase-2	GSMUA_Achr10P16420_001_MUSAC	Detected only in inoculated	0.01	38.77	2
Nitrogen compound					
Dihydrolipoyllysine-residue acetyltransferase component 2 of pyruvate dehydrogenase (mitochondrial-like)	GSMUA_Achr10P08050_001_MUSAC	-1.8	0.04	64.44	3
Primary metabolic process					
30-dai					
Probable lipoxygenase (LOX) 4	GSMUA_Achr1P22970_001_MUSAC	-2.3	0.00	43.71	1
60-dai					
T-complex protein 1 subunit zeta-like	GSMUA_Achr1P14710_001_MUSAC	Detected only in inoculated	0.01	33.21	2
Ru large subunit-binding protein subunit alpha	ITC1587_BchrUn_random_P35868_MUSBA	Detected only in inoculated	0	48.58	1
Fructokinase-1-like	GSMUA_Achr11P11150_001_MUSAC	829.8	0	63.94	4
26s proteasome regulatory subunit 4 homolog A	ITC1587_Bchr4_P08913_MUSBA	24.2	0.02	80.85	1
Ru large subunit-binding protein subunit (chloroplastic-like)	GSMUA_Achr9P23240_001_MUSAC	12.3	0.04	61.06	2
26s protease regulatory subunit 6b homolog	ITC1587_Bchr7_P20965_MUSBA	9.6	0.03	37.6	1
Heat shock 70 kDa (mitochondrial-like)	GSMUA_Achr3P12480_001_MUSAC	2.5	0.05	38.53	2
ATP-dependent clp protease ATP -binding subunit CLPA homolog chloroplastic	GSMUA_AchrUn_randomP22440_001_MUSAC	-2.4	0.04	53.63	1
Malate mitochondrial-like	GSMUA_Achr4P08580_001_MUSAC	-2.2	0.01	71.87	3
Mitochondrial-processing peptidase subunit alpha-like	GSMUA_Achr7P13650_001_MUSAC	-2.3	0.01	55.11	1
Peptidyl-prolyl cis-trans isomerase cyp95-like	ITC1587_Bchr10_P31266_MUSBA	-2.5	0.01	38.12	1
Pi-plc x domain-containing protein at5g67130-like	GSMUA_Achr6P25660_001_MUSAC	-3	0.01	36.74	1
Pi-plc x domain-containing protein at5g67130-like	ITC1587_Bchr6_P16564_MUSBA	-7.4	0	31.96	2
Proteolysis					
60-dai					
Probable mitochondrial-processing peptidase subunit beta	GSMUA_Achr7P00560_001_MUSAC	-1.7	0.03	54.91	6
Responses					
60-dai					
Abiotic stimulus					
70 kda peptidyl-prolyl isomerase-like	GSMUA_AchrUn_randomP02470_001_MUSAC	Detected only in inoculated	0	35.39	2
CBS domain-containing protein (mitochondrial-like)	GSMUA_Achr3P26630_001_MUSAC	-2.4	0.01	43.38	1
Aconitate (cytoplasmic-like)	GSMUA_Achr11P01170_001_MUSAC	-2.5	0.01	34.2	2
Probable succinyl- ligase	ITC1587_Bchr2_P04196_MUSBA	-2.4	0.02	48.1	2
Succinyl- ligase	GSMUA_Achr8P19050_001_MUSAC	-2.8	0	71.87	5
Biotic stimulus					
Pathogenesis-related protein 1-like	ITC1587_Bchr9_P26466_MUSBA	-30	0.02	61.68	1
Endogenous stimulus					
Alpha-galactosidase	GSMUA_Achr6P15820_001_MUSAC	-4.3	0.03	34.19	1
ROS					
Germin-like protein 5–1	GSMUA_Achr1P25160_001_MUSAC	-2.7	0.05	46.23	1
Stress					
Probable l-ascorbate peroxidase (chloroplastic)	GSMUA_Achr10P16040_001_MUSAC	-5.4	0.03	31.3	1
Peroxidase 5-like	GSMUA_Achr8P12370_001_MUSAC	-8.2	0.01	60.05	2
Single organism process					
60-dai					
Putative uncharacterised protein	GSMUA_Achr11P04110_001_MUSAC	-2.7	0.02	61.8	1
Transporter activity					
60-dai					
Carrier protein (mitochondrial-like)	ITC1587_Bchr8_P24300_MUSBA	Detected only in inoculated	0.01	35	1
Cytochrome C oxidase subunit 6b-1-like	GSMUA_Achr7P11740_001_MUSAC	-2.2	0	30.35	1
No Gene Ontology					
30-dai					
Fasciclin-like arabinogalactan protein 2	GSMUA_Achr2P05440_001_MUSAC	Not detected in inoculated sample	0.01	45.86	1
60-dai					
Enoyl	GSMUA_Achr1P19640_001_MUSAC	Detected only in inoculated	0.01	49.35	1
Putative Sec12-like protein 2	GSMUA_Achr6P10220_001_MUSAC	Detected only in inoculated	0.01	38.02	1
Isoflavone reductase-like protein	GSMUA_Achr2P14320_001_MUSAC	10.3	0.04	77.33	6
btb poz domain-containing protein at5g60050	ITC1587_Bchr9_P28128_MUSBA	6.5	0	51.86	2
Heat shock cognate 70 kda protein 2-like	GSMUA_Achr9P03960_001_MUSAC	4.2	0	31.8	1
40S ribosomal protein S27-2	GSMUA_Achr3P18600_001_MUSAC	3.7	0.03	34.9	1
Protein binding protein	GSMUA_Achr8P15600_001_MUSAC	3	0.01	34.51	1
mfp1 attachment factor 1-like	GSMUA_Achr4P14260_001_MUSAC	-2.2	0	50.88	1
Md-2-related lipid recognition domain-containing protein	ITC1587_Bchr5_P14231_MUSBA	-2.8	0.03	58.58	1
Seed specific protein Bn15D1B, putative, expressed	GSMUA_Achr8P13580_001_MUSAC	-5.2	0.01	37.06	1
PREDICTED: uncharacterised protein LOC103996731	GSMUA_Achr9P02000_001_MUSAC	-5.7	0.01	31.72	1

### Proteome profiling of *Meloidogyne incognita*–inoculated Grand naine root fragments

**Fig 3:**
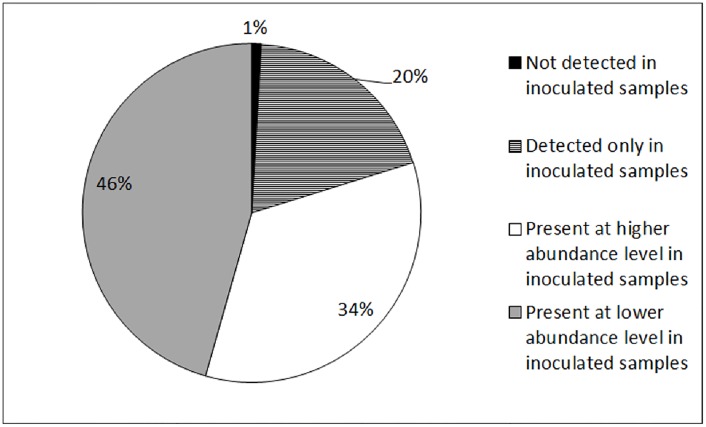
Pie chart showing the percentage of 114 proteins with significant abundance difference in inoculated samples when compared to control samples at both 30-dai and 60-dai time points.

**Fig 4:**
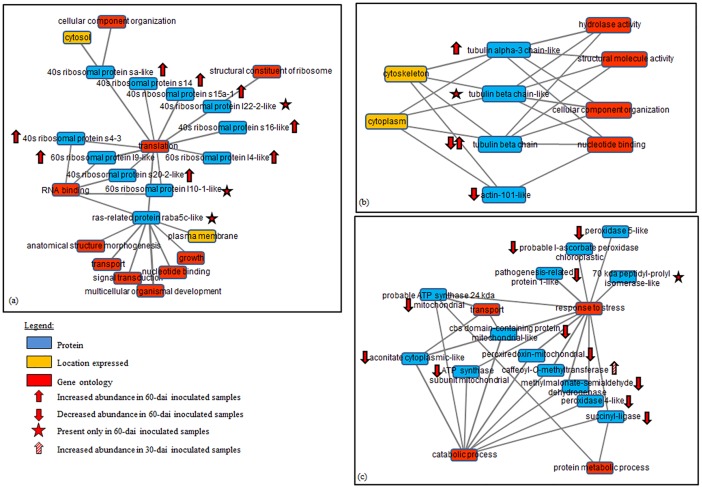
Protein-GO interaction profile of banana root proteome generated from Cytoscape showing (a) selected proteins involved in fundamental biological processes that were either present at higher abundance level or detected only in inoculated samples, (b) proteins involved in cellular component organisation that were present at different abundance levels which were also involved in three other biological functions, and (c) all proteins involved in response to stresses that were present in lower abundances in inoculated samples except for peptidyl-prolyl isomerase-like (at 60-dai) and caffeoyl-*O*-methyltransferase (at 30-dai). These stress proteins were also involved in transport, catabolic and protein metabolic processes.

Ninety-six percent of 114 proteins that showed significant abundance difference belong to the 60T vs. 60C matrix while the remainder belongs to the 30T vs. 30C matrix. The majority of the proteins (54%; inclusive of those detected only in inoculated samples) were present at higher abundance level while 46% were present at lower abundance level in inoculated samples (Fig 3). Proteins involved in *M*. *incognita*-Grand naine interaction were further categorised into 17 groups based on their annotated gene ontologies (GO) as described in Table 1. Cytoscape analyses based on shared GOs revealed interactions amongst 114 banana root proteins occurring during nematode infestation (Fig 4a–4c). We further investigated protein-GO interactions involved in fundamental biological process, cellular component organisation and response to stresses.

#### Fundamental biological processes

Interaction of ribosomal proteins categorised under the fundamental biological processes (Table 1) was investigated as all of the proteins were either detected only in inoculated samples or present at higher abundance level in inoculated samples. We found that these proteins shared a similar function in translation (Fig 4a). Three ribosomal proteins namely 40S ribosomal protein s4-3 (GSMUA_Achr2P01640_001_MUSAC), 60S ribosomal protein I10a-1-like (ITC1587_Bchr3_P07546_MUSBA) and 40S ribosomal protein s20-2-like (ITC1587_Bchr3_P06192_MUSBA) were mapped to be involved in RNA binding while 40S ribosomal protein sa-like (GSMUA_Achr1P21820_001_MUSAC) were involved in cellular component organisation. A protein categorised in catalytic and hydrolytic group namely Ras-related protein raba5c-like (ITC1587_Bchr11_P33367_MUSBA; Table 1) that was detected only in inoculated samples (Fig 4a) shared the same function as these ribosomal proteins in translation and RNA binding.

#### Cellular component organization

We noted that proteins involved in cellular component organisation shared similar annotations with four other proteins that were involved in translation factor activity and cellular protein modification process. Twelve proteins that are involved in cellular component organisation showed differential abundance levels. Two of the proteins namely Ru large subunit-binding protein subunit alpha (ITC1587_BchrUn_random_P35868_MUSBA) and tubulin beta chain-like (ITC1587_BchrUn_random_P35428_MUSBA) were detected only in inoculated samples. On the other hand, alpha-glucan-protein synthase (ITC1587_Bchr4_P10810_MUSBA), tubulin alpha-3-chain-like (ITC1587_Bchr6_P17875_MUSBA) and 40S ribosomal protein Sa-like protein (GSMUA_Achr1P21820_001_MUSAC) showed higher abundance levels in inoculated samples while other proteins were present in lower abundances in inoculated samples (Fig 4b). Interestingly, we had profiled two tubulin beta chain protein species (ITC1587_Bchr6_P16601_MUSBA and GSMUA_Achr6P04600_001_MUSAC) involved in cellular component organisation that showed differing abundance levels in inoculated samples. When the interaction of the three tubulin proteins was selected, it was revealed that these proteins shared nucleotide binding function with actin101-like protein (GSMUA_Achr10P03730_001_MUSAC) that, according to GO analysis, was present in cytoplasm and cytoskeleton (Fig 4b).

#### Stress responses

Thirteen proteins were found to be involved in stress responses. Of these, one was present only in inoculated root tissues at 60-dai and the remaining twelve were present at lower abundance level in the same samples. On the other hand, at 30-dai caffeoyl-*O*-methyltransferase (GSMUA_Achr6P36400_001_MUSAC) was present at higher abundance level in inoculated samples compared to the control ones (Fig 4c). The interaction of these proteins were further investigated and we found that only four of these proteins namely pathogenesis related protein 1-like (ITC1587_Bchr9_P26466_MUSBA), chloroplastic probable 1-ascorbate peroxidase (GSMUA_Achr10P16040_001_MUSAC), peroxidase 5-like (GSMUA_Achr8P12370_001_MUSAC) and 70kDa peptidyl-prolyl isomerase-like (GSMUA_AchrUn_randomP02470_001_MUSAC) were annotated to be solely involved in response to stress. CBS domain-containing protein (mitochondrial-like) (GSMUA_Achr3P26630_001_MUSAC) is involved in two other functions namely catabolic process and transportation, and share similar annotation with probable ATP synthase 24 kDa (mitochondrial; GSMUA_Achr6P02850_001_MUSAC) and cytoplasmic-like aconitate (GSMUA_Achr11P01170_001_MUSAC) for the same functions. On the other hand, the remaining five proteins mapped showed involvement in catabolic processes.

### KEGG pathways associated with nematode infection in banana root samples

We investigated three KEGG pathways that were associated with defence and giant cell maintenance in bananas which include lignification and energy metabolism namely phenylpropanoid biosynthesis, glycolysis and citrate cycle pathways.

#### Lignification

**Fig 5:**
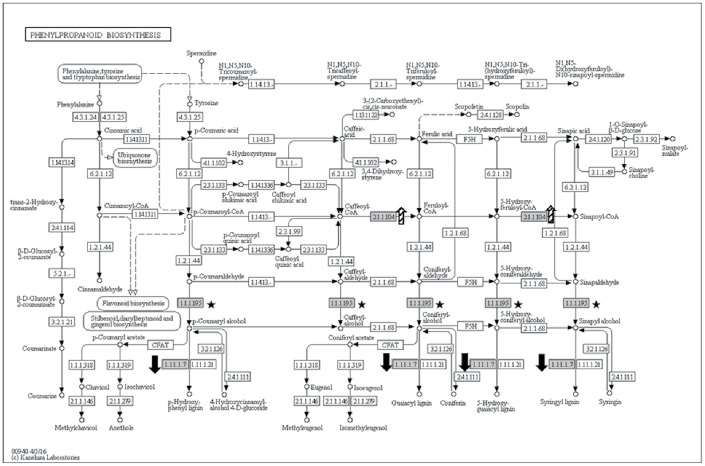
Phenylpropanoid biosynthesis KEGG pathway showing three differentially abundant enzymes (grey boxes) in the inoculated samples at 30-dai (arrows with slanted lines) and 60-dai (black arrows). Caffeoyl-*O*-methyltransferase (EC 2.1.1.104; GSMUA_Achr6P36400_001_MUSAC) was present 5.3x higher in abundance in inoculated samples. At 60-dai, cinnamyl alcohol dehydrogenase (CAD; EC 1.1.1.195; GSMUA_Achr4P06150_001_MUSAC; black stars) was detected only in inoculated samples and lignin-forming anionic peroxidase-like (EC1.11.1.7; GSMUA_Achr4P05250_001_MUSAC; black arrows) was present 4.1x lesser in abundance in inoculated samples.

Lignification process is greatly influenced by enzyme activities in phenylpropanoid biosynthesis and three enzymes were found to be implicated in this pathway (Fig 5). Here, we found that caffeoyl-*O*-methyltransferase (EC 2.1.1.104; GSMUA_Achr6P36400_001_MUSAC) was present at an increased abundance level (5.3-fold) at 30-dai in inoculated samples. Cinnamyl alcohol dehydrogenase (CAD; EC 1.1.1.195; GSMUA_Achr4P06150_001_MUSAC), on the other hand, was detected only in inoculated samples at 60-dai. At the same time point, lignin-forming anionic peroxidase-like enzyme (EC 1.11.1.7; GSMUA_Achr4P05250_001_MUSAC) was found present at low abundance level (4.1-fold) in inoculated samples.

#### Carbohydrate catabolism

**Fig 6:**
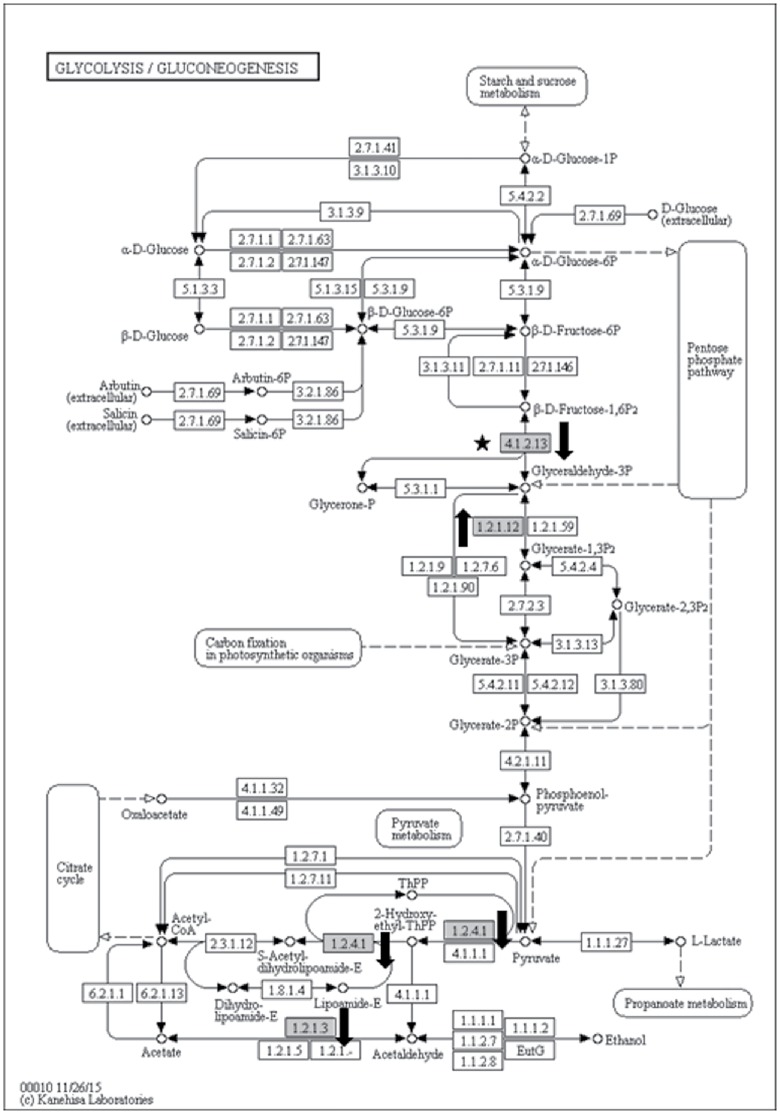
Glycolysis/Gluconeogenesis KEGG pathway showing four differentially abundant enzymes (grey boxes) in the inoculated samples at 60-dai. Two putatively different protein species of fructose-bisphosphate aldolase (EC 4.1.2.13) were recovered in this study with each showing differential abundances at 60-dai. One isoform (ITC1587_Bchr5_P14394_MUSBA) was present at lower abundance level (-5.1x) in inoculated root samples while another (ITC1587_Bchr8_P21572_MUSBA) was detected only in inoculated samples (black star). Glyceraldehyde-3-phosphate dehydrogenase (EC 1.2.1.12; GSMUA_Achr5P25410_001_MUSAC) was present at 469-fold higher in inoculated root samples with another two enzymes namely aldehyde dehydrogenase (EC 1.2.1.3; GSMUA_AchrUn_randomP11080_001_MUSAC) and pyruvate dehydrogenase (EC 1.2.4.1; GSMUA_Achr5P25000_001_MUSAC) were present at lower abundance levels in inoculated samples (-5.8x and -3.7, respectively). Enzymes present in lower abundance level were marked with black arrows pointing downward while the ones present in higher abundance level were marked with black arrows pointing upward.

Four proteins were differentially abundant in the glycolysis pathway (Fig 6). Interestingly, two putative protein species of fructose-bisphosphate aldolase (EC 4.1.2.13; ITC1587_Bchr5_P14394_MUSBA and ITC1587_Bchr8_P21572_MUSBA) were recovered in this study with each showing differing abundance levels at 60-dai. The putative isoform with the accession number ITC1587_Bchr5_P14394_MUSBA was present at lower abundance level (5.1-fold) while another putative isoform (ITC1587_Bchr8_P21572_MUSBA) was detected only in inoculated samples. This enzyme together with glyceraldehyde-3-phosphate dehydrogenase (EC 1.2.1.12; GSMUA_Achr5P25410_001_MUSAC) that showed significantly increased abundance level (468.9-fold) are involved in the production of glycerate-3-phosphate. On the other hand, aldehyde dehydrogenase (EC 1.2.1.3; GSMUA_AchrUn_randomP11080_001_MUSAC) and pyruvate dehydrogenase (EC 1.2.4.1; GSMUA_Achr5P25000_001_MUSAC) were present at lower abundance levels in inoculated samples at 5.8-fold and 3.7-fold, respectively.

#### Energy metabolism

**Fig 7:**
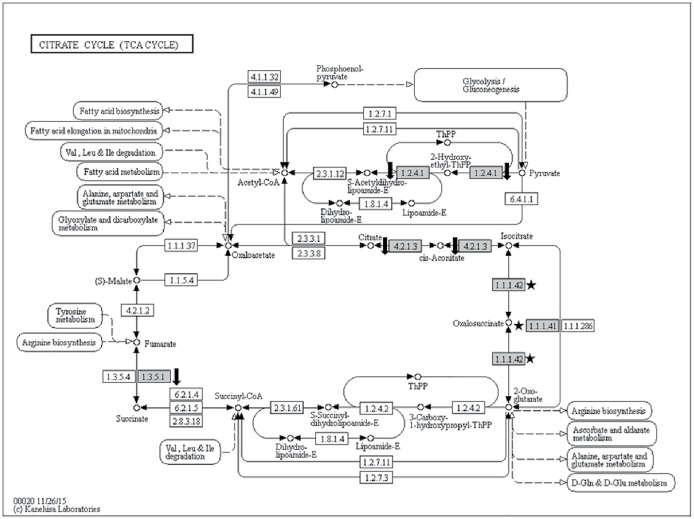
Citrate (TCA) cycle KEGG pathway showing four differentially abundant enzymes (grey boxes) in the inoculated samples at 60-dai. An enzyme responsible for conversion from pyruvate to acetyl-CoA, Pyruvate dehydrogenase (EC 1.2.4.1; GSMUA_Achr5P25000_001_MUSAC) was present 3.7-fold lower in abundance in inoculated samples. Aconitate (EC 4.2.1.3; GSMUA_Achr11P01170_001_MUSAC) and succinate dehydrogenase (EC 1.3.5.1; GSMUA_Achr6P31640_001_MUSAC and ITC1587_Bchr7_P18621_MUSBA) too, was present in lower abundance level in inoculated samples. Two types (EC 1.1.1.41 and EC 1.1.1.42) of isocitrate dehydrogenase (GSMUA_Achr1P05110_001_MUSAC) were detected only in inoculated samples (marked with black stars). Enzymes present in lower abundance levels in inoculated samples were marked with black arrows pointing downward.

Four enzymes were present at differential abundances in the Citrate cycle pathway (Fig 7). From the four, three were present at lower abundance level in inoculated samples. The three enzymes are aconitate (EC 4.2.1.3; GSMUA_Achr11P01170_001_MUSAC), pyruvate dehydrogenase e1 component subunit beta (EC 1.2.4.1; GSMUA_Achr5P25000_001_MUSAC) and succinate dehydrogenase (EC 1.3.5.1; GSMUA_Achr6P31640_001_MUSAC; ITC1587_Bchr7_P18621_MUSBA). Two types (EC 1.1.1.41 and EC 1.1.1.42) of isocitrate dehydrogenase (GSMUA_Achr1P05110_001_MUSAC) were detected only in inoculated samples.

## Discussion

In this study, we investigated banana responses at the 30^th^—and 60^th^- day after inoculation (dai) with *Meloidogyne incognita* using a Liquid Chromatography-Mass Spectrometry (LC-MS) shotgun approach. We opted to use our in-house-designed single inoculation site approach [35] due to our past failures in obtaining consistent data using a general inoculation approach described by [41]. We inoculated 1000 J_2_ *M*. *incognita* on approximately 50 mg root fragment in order to increase the number of infected cells per 50 mg root fragment (wet weight used in our protein isolation procedure). By doing so, we hypothesised that the chance to detect protein abundance changes in two sample types (inoculated vs. control) will also be increased. This approach was deemed efficient since galls were visible to the naked eye as swellings on inoculated banana roots for both time points i.e. 30- and 60-dai (Fig 1). It is noteworthy that galls were more visible on inoculated samples at 60-dai compared to 30-dai. This may be due to nematode population build-up in 60-day old root fragments. In addition, we have also successfully identified 114 proteins (from A and B genomes) showing significant abundance difference (p ≤ 0.05) between inoculated and non-inoculated samples. We compared our data with the transcriptomic data recently published by Castaňeda et al. [42] and found that 71 A genome banana genes obtained in our study out of 77 obtained in [42] were significantly modulated upon *M*. *incognita* inoculation in susceptible banana varieties namely 4279–06 and Cavendish (CAV) at early time points (S1 Table). Such a high level of match (92%) between transcriptomics and proteomics data substantiates the involvement of these 71 genes in banana-nematode interaction.

Our result implied that a major reprogramming of plant metabolism had taken place during *M*. *incognita*—Grand naine interaction. The global proteome profile obtained in this study illustrated an incidence of a battle involving attack and defence occurring between the two organisms during pathogen colonisation in the root fragments leading to a compatible interaction. Gheysen and Mitchum [43] stated that molecular mimicry and manipulative behaviour of nematode effector molecules reprogrammed plant cells to form feeding cells and simultaneously suppress host stress and defence responses. To counter this infection, generally, plants have evolved two recognition mechanisms namely the transmembrane pattern recognition receptor (PRR), and the intracellular immune receptors known as resistance (R) proteins [44]. The former responds to microbial/pathogen—associated molecular patterns (MAMPS or PAMPS) inducing PAMP-triggered immunity (PTI) while the latter responds to effector molecules inducing effector-triggered immunity (ETI) [44, 45]. With regards to ETI, effector molecules secreted by the pathogen will be recognised by specific *resistance* (*R*) genes in plants and cascades of defence mechanisms will be triggered. This includes the activation of hypersensitive responses (HR) in cells adjacent to the infection site. Once HR is activated, plant’s systemic acquired resistance (SAR) pathway will eventually be turned on. Although we found that the majority of proteins involved in responses (Table 1; Fig 4c) were present in lower abundances in inoculated samples, the involvement of R protein in Grand naine—*M*. *incognita* interaction was not captured in this experiment. Conversely, Castaňeda et al. [42] had demonstrated that three potential *R* genes namely LRR receptor-like serine/threonine-protein kinases (GSMUA_Achr3G10820_001, GSMUA_Achr1G01110_001 and GSMUA_Achr3G28550_001) were significantly upregulated at 3-dai in susceptible banana cultivars. These genes were then downregulated at 10-dai, which may justify the inability of the LC-MS platform to detect its significant abundance difference in the root tissues at 30- and 60-dai.

A calcium influx- signalled oxidative burst [46, 47] producing reactive oxygen intermediates occurs soon after host plant-pathogen contact [25, 48]. The activation of these intermediates results in the activation of defence gene expression in adjacent cells triggering HR, a form of programmed cell death in plants. This includes cell wall reinforcement such as lignification occurring only at the site of infection. Both Levine et al. [47] and Backiyarani et al. [49] agreed that cell wall fortification was one of the defence mechanisms included in HR. Lignification is a defining feature of secondary cell wall formation. Besides its essential role in maintaining cell rigidity [50], lignin plays an important role in plant disease resistance [51]. Lignin biosynthesis was reported to be induced upon wounding, pathogen infection, metabolic stress and perturbations in cell wall structure [50]. Since lignin is a complex phenylpropanoid polymer [52], its synthesis is greatly influenced by enzyme activities in phenylpropanoid biosynthesis pathway (Fig 5). In our study, at 30-dai, a protein that participates at an earlier stage of lignin production pathway namely caffeoyl-*O*-methyltransferase (GSMUA_Achr6P36400_001_MUSAC; EC 2.1.1.104) [52, 53] was found at a significantly high abundance level (5.3 folds) in inoculated samples compared to control samples. Our data corroborate that of published in Castaňeda et al. [42] whom demonstrated that caffeoyl-*O*-methyltransferase (GSMUA_Achr6P36400_001_MUSAC) was downregulated at 3-dai in 4379–06 and 7-dai in CAV cultivars upon *M*. *incognita* inoculation and was later upregulated at a later stage of infection suggesting successful colonisation. At 60-dai, we found cinnamyl alcohol dehydrogenase (CAD; GSMUA_Achr4P06150_001_MUSAC; EC 1.1.1.195)–an important enzyme catalysing lignin formation [54] to be detected only in inoculated samples, which may indicate a defence response. However since the abundance level of lignin-forming anionic peroxidase-like protein (GSMUA_Achr4P05250_001_MUSAC; EC 1.11.1.7) was four-fold lower in the same samples at the same time point, lignification level was expected to be reduced in these samples. Vanholme et al. [50] reported that the down-regulation of CAD increases the incorporation of cinnamaldehydes into lignin polymer. This suggests that the increased level of CAD at 60-dai could potentially decreases the incorporation of cinnamaldehydes into the polymer in the inoculated samples, indicating that lignification was halted at 60-dai. This suggests that at this time point, *M*. *incognita* might have targeted CAD for giant cell formation/maintenance which involves cell wall dissolution. It is noteworthy that Glazer et al. [55] had reported inhibition of xylem lignification in *Meloidogyne javanica*-infected tomato roots at 14- and 28-dai. We hypothesise that in an interaction involving banana and root-knot nematodes, such an increase in protein abundance of a host at a later stage of infection (60-dai) may translate to manipulation of host’s cell system by the nematodes in order to maintain their nutrient sink, the giant cells. This is in line with that of found in Castaňeda et al. [42] highlighting the decreased level of expression of cell wall-associated genes in nematode- inoculated root tissues over time. In addition to that, we also observed that a probable calcium-binding protein, calmodulin-like protein 7 (CML7) (ITC1587_Bchr9_P27746_MUSBA) was present at 5.2 folds higher in abundance in inoculated samples at 60-dai (Table 1) implying that the influx of these second messengers (Ca^2+^ ions) still occurred in the infected cells even at a later stage of infection. This suggests that signalling and major biochemical changes are still occurring in the nematode-inoculated banana roots at 60-dai. It is noteworthy that Castaňeda et al. [42] demonstrated that another calcium binding protein, CML 18 was significantly upregulated in an *M*. *incognita*- inoculated Cavendish banana variety at 10-dai.

**Fig 8:**
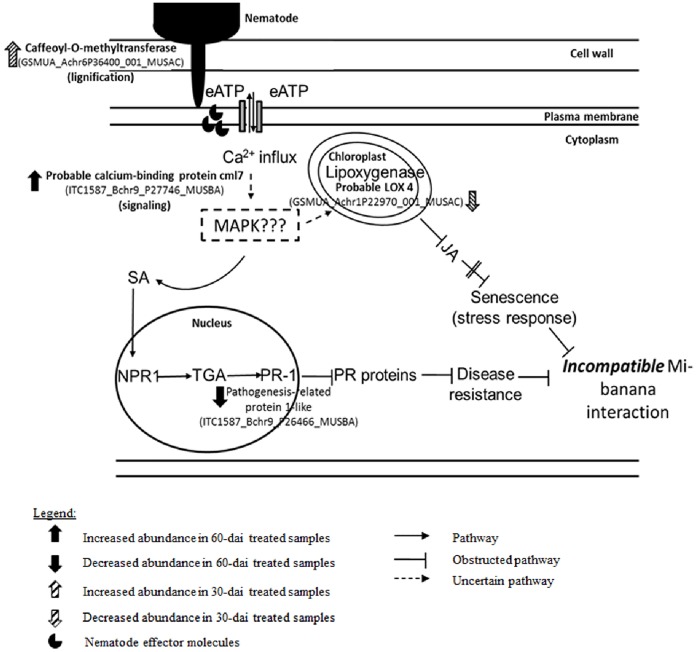
A hypothetical schematic defence pathway proposed on a compatible interaction between *M*. *incognita* and Grand naine primarily based on Castaňeda et al. [42], Hahlbrock et al. [46], Levine et al. [47], and Tanaka et al. [71]. Nematode stylet penetration into the plant cell results in production of extracellular adenosine-5’- triphosphate (eATP) which signals for calcium (Ca^2+^) influx. Such a signalling response will trigger the stress and disease resistance pathway leading to incompatibility. Both pathways were obtained from plant and hormone signal transduction KEGG pathways (http://www.genome.jp/kegg-bin/show_pathway?ath04075). Note that the pathway leading to cell senescent was abridged for the purpose of simplicity. Proteins involved in stress response and disease resistance pathways obtained in our study are mapped here. *Mitogen-activated protein kinase* (*MAPK*); *Nonexpresser of PR* genes 1 (*NPR*1); Pathogenesis-related protein 1 (PR-1).

We also found a probable lipoxygenase (LOX) 4 protein (GSMUA_Achr1P22970_001_MUSAC), to be present at two folds lower in abundance in inoculated samples at 30-dai. This gene was found to be significantly downregulated at the transcript level as early as 3-dai [42]. LOX catalyses the oxygenation of fatty acids involved in the production of jasmonic acid (JA) [56], the signalling molecules that modulate HR. Tremblay et al. [57] reported that *LOX2* gene expression was suppressed during a compatible *Glycine max*-*Phakopsora pachyrhizi* interaction while Ibrahim et al. [1] found that the expression of genes encoding enzymes of the alpha-linolenic acid pathway leading to jasmonic acid production in *Glycine max—M*. *incognita* interaction was turned off after a prolonged infection (10-weeks after inoculation). This led us to echo the suggestion made by Ibrahim et al. [1] that genes involved in JA synthesis could be targets for further testing for conferment of nematode resistance in bananas by overexpressing them. Since the expression of a key gene correlated to HR had been turned down at 30 dai, our result showed that the subsequent cascade leading to plant resistance has been ‘battered’ (Fig 8). Our analysis revealed that pathogenesis-related protein 1-like (PR1-like; ITC1587_Bchr9_P26466_MUSBA) was present 30-fold lesser in inoculated samples at 60-dai. This implies that SAR pathway is shut down upon *M*. *incognita* inoculation in Cavendish bananas. Spoel and Dong [58] motioned that accumulation of salicylic acid (SA) induces the gene expression pathway leading to the production of PR proteins. Such a signal transduction cascade will result in an establishment of immune memories leading to SAR. From our result, we hypothesise that the nematodes may have successfully ‘tampered’ with Grand naine immunity system at least at 30-dai or much earlier.

*M*. *incognita* infection not only impinged upon plant defence system but also manipulated the cell division cycle in order to re-design root morphology to form galls. This re-designing involves the induction of multiple rounds of acytokinetic mitosis amongst the infected cells to form multinucleated giant cells [1, 16, 43] that serve as nutrient sinks for the nematodes [59]. Although we did not capture the involvement of cyclin-dependent kinases (CDKs) as that of reported by de Almeida Engler et al. [15] in our data, consistent with the presence of gall-forming multinucleated giant cells observed, we have successfully profiled proteins involved in cellular organisation (Fig 4b) of the inoculated root samples. In agreement with that of reported by de Almeida Engler et al. [60], our analysis shows that three tubulin proteins (Table 1) were found to be present at high abundance levels in inoculated samples reflecting that a major cytoskeletal rearrangement had taken place in these samples. Note that both α- and β- tubulin proteins will polymerise into microtubules—one of the key components of the cytoskeletons that play an indirect role in maintaining cell shape [61]. Interestingly, one of the tubulin-beta chain protein species obtained (GSMUA_Achr6P04600_001_MUSAC) was present at lower abundance in inoculated sample while the other (ITC1587_BchrUn_random_P35428_MUSBA) was present at ~200-fold higher in the same tissues suggesting that one of these protein species is important in microtubule formation of giant cells. In 2004, de Almeida Engler et al. [60] reported the presence of a large number of unusual and randomly oriented actin bundles and cables during giant cell development. We found that the abundance of an actin-101-like protein (GSMUA_Achr10P03730_001_MUSAC) was decreased by almost two-fold in inoculated samples suggesting abnormal cell development had occurred in inoculated samples. This may justify the importance of actin for a successful *M*. *incognita* colonisation in plant root tissues. It is noteworthy that the transcriptomic expression profile of both tubulin-beta chain (GSMUA_Achr6P04600_001_MUSAC) and actin (GSMUA_Achr10P03730_001_MUSAC) genes obtained in Castaňeda et al. [42] at earlier nematode infection time-points matched our proteome profiling data.

A total of 11 ribosomal proteins were found either at higher abundances or detected only in inoculated samples. This finding is consistent with that of reported by Jammes et al. [13] where 71 genes encoding 40S and 60S ribosomal proteins were specifically upregulated in *M*. *incognita*- inoculated Arabidopsis root samples suggesting an increased level of protein synthesis in giant cells. This justified the notion proposed by Ibrahim et al. [1] that nematodes utilise plant resources to develop and reproduce. In our study, proteins involved in carbohydrate catabolism and energy metabolism were differentially abundant in inoculated root samples to either meet the nematode’s energy and carbon demand, or, evading plant defense response. We found potentially two fructose-bisphosphate aldolase (4.1.2.13) protein species present at opposing abundance levels in glycolysis/gluconeogenesis KEGG pathway (Fig 5). This enzyme catalyses an aldol cleavage of fructose-1, 6-bisphosphate to dihydroxyacetone-phosphate and glyceraldehyde 3-phosphate in a reversible aldol condensation [62]. It is noteworthy that, this aldolase could either be cytosolic or chloroplastic in plants [63]. Although both protein species differ in their primary structure and intracellular localisation, they both play an essential role in carbohydrate metabolism; hence, differing levels of abundance of both protein species could implicate plant growth [64]. Konishi et al. [64] reported that they observed notable accumulation of Gibberellic acid-induced aldolase at apical region of rice seedling roots. Interestingly, active cell division occurred in both apical region and root differentiation zone where the nematodes reside [16] suggesting that increased levels of aldolase may stimulate root cell growth that mediate energy production. However, in the case of nematode-infested root system, we hypothesise that cell growth, in this context, reflects giant cell formation, and plant energy production may be manipulated by the pathogen to sustain their longevity. Another enzyme in the glycolytic pathway present at significantly high abundance level was glyceraldehyde-3-phosphate dehydrogenase (GAPDH; EC 1.2.1.12; GSMUA_Achr5P25410_001_MUSAC). Castaňeda et al. [42] demonstrated that this gene was upregulated as early as 10-dai in a Cavendish banana upon *M*. *incognita* infestation. This enzyme is responsible to produce energy and supply intermediates for cellular metabolism [65] and had been implicated in embryo development, root growth [66] as well as in transducing hydrogen peroxide signals in Arabidopsis response to stress [67]. We also noted that two enzymes involved in glycolysis namely pyruvate dehydrogenase (EC 1.2.4.1; GSMUA_Achr5P25000_001_MUSAC) and aldehyde dehydrogenase (EC 1.2.1.3; GSMUA_AchrUn_randomP11080_001_MUSAC), responsible in the production of acetyl-CoA, were present at lower abundance level in inoculated samples suggesting implicated acetyl-CoA production in inoculated samples. Acetyl-CoA is an important molecule involves in the Citrate cycle (TCA cycle) pertinent in ATP production [61]. We found four enzymes involved in the Citrate cycle pathway to be differentially abundant. Although isocitrate dehydrogenase (EC 1.1.1.41 and EC 1.1.1.42; GSMUA_Achr1P05110_001_MUSAC) was found to be present only in inoculated sample, an enzyme that was responsible in converting succinate to fumarate namely succinate dehydrogenase (EC 1.3.5.1; GSMUA_Achr6P31640_001_MUSAC; ITC1587_Bchr7_P18621_MUSBA) was present twice times lower in abundance in inoculated samples. This may potentially implicate the production of ATP in the inoculated samples. Our result showed four ATP synthase subunits (GSMUA_AchrUn_randomP15230_001_MUSAC, GSMUA_Achr10P27350_001_MUSAC, GSMUA_Achr9P21710_001_MUSAC and ITC1587_Bchr10_P31293_MUSBA) and one probable mitochondrial ATP synthase (GSMUA_Achr6P02850_001_MUSAC) to be present in lower abundance levels (1.5 to 2.6 folds lower; Table 1) in inoculated samples. ATP synthase is known as a highly conserved enzyme that involved in catalysing ATP synthesis from ADP and phosphate during mitochondrial respiration [68]. Although it was expected that the abundance level for ATP synthase to be high in tissues undergoing adverse metabolic reprogramming activity such as the galls, our profiling result proved otherwise. This enzyme is a complicated protein complex and is divided into two sectors namely a soluble globular F_1_ catalytic sector and a membrane-bound F_0_ proton-translocating sector [69]. In our result, one of the proteins of the latter complex was present at a lower abundance level in inoculated samples suggesting the presence of deformed ATP synthase complex with an inability to translocate proton in inoculated root cells. Proton translocation is important for the cell to generate proton electrochemical gradient that serves as the driving force for ion and metabolite uptake across the plasma membrane [70]. Therefore the deformation of ATP synthase complex in the inoculated samples may indicate interference in such mechanism in nematode-infested banana root cells. This set-back will eventually impair plant growth and fruit production.

In this study, by using our in-house-designed single inoculation site approach [35] coupled with an Orbitrap LC-MS platform, we conclude that 114 banana root proteins showed significant abundance changes when inoculated with *Meloidogyne incognita* at 30- and 60-dai. Our study revealed that these changes affected proteins involved primarily in fundamental biological processes, cellular component organisation and stress responses. Our analysis provided a new spectrum of knowledge especially on plant-parasite interaction from the perspective of a non-model organism. Corroborating transcriptomic data obtained in [42], we had identified players in banana defence and response pathway against *M*. *incognita* infestation namely caffeoyl- O-methyltransferase (GSMUA_Achr6P36400_001_MUSAC) probable lipoxygenase (LOX) 4 protein (GSMUA_Achr1P22970_001_MUSAC) and pathogenesis-related protein 1-like (PR1-like; ITC1587_Bchr9_P26466_MUSBA) that could serve as targets for further functional tests in order to develop a tolerant/ resistant banana cultivar against *M*. *incognita*. Here, we propose a hypothetical defence pathway leading to a compatible interaction between Grand naine—*M*. *incognita* primarily based on Castaňeda et al. [42], Hahlbrock et al. [46], Levine [47], and Tanaka et al. [71] (Fig 8). We also have mapped the stress and defence proteins obtained from our study to this hypothetical pathway.

## Supporting information

S1 Table Comparison between transcriptomic data obtained in [42] and proteomic data from the current study revealed that 71 A genome banana genes in the current study out of 77 obtained in [42] were significantly modulated during banana-*Meloidogyne incognita* interaction. (XLSX)
